# Nociceptors as chronic drivers of pain and hyperreflexia after spinal cord injury: an adaptive-maladaptive hyperfunctional state hypothesis

**DOI:** 10.3389/fphys.2012.00309

**Published:** 2012-08-02

**Authors:** Edgar T. Walters

**Affiliations:** Department of Integrative Biology and Pharmacology, University of Texas Medical School at HoustonHouston, TX, USA

**Keywords:** inflammatory pain, neuropathic pain, primary afferent neuron, hyperexcitability, sensitization, memory of injury, evolution

## Abstract

Spinal cord injury (SCI) causes chronic peripheral sensitization of nociceptors and persistent generation of spontaneous action potentials (SA) in peripheral branches and the somata of hyperexcitable nociceptors within dorsal root ganglia (DRG). Here it is proposed that SCI triggers in numerous nociceptors a persistent hyperfunctional state (peripheral, synaptic, and somal) that originally evolved as an adaptive response to compensate for loss of sensory terminals after severe but survivable peripheral injury. In this hypothesis, nociceptor somata monitor the status of their own receptive field and the rest of the body by integrating signals received by their peripheral and central branches and the soma itself. A nociceptor switches into a potentially permanent hyperfunctional state when central neural, glial, and inflammatory signal combinations are detected that indicate extensive peripheral injury. Similar signal combinations are produced by SCI and disseminated widely to uninjured as well as injured nociceptors. This paper focuses on the uninjured nociceptors that are altered by SCI. Enhanced activity generated in below-level nociceptors promotes below-level central sensitization, somatic and autonomic hyperreflexia, and visceral dysfunction. If sufficient ascending fibers survive, enhanced activity in below-level nociceptors contributes to below-level pain. Nociceptor activity generated above the injury level contributes to at- and above-level sensitization and pain (evoked and spontaneous). Thus, SCI triggers a potent nociceptor state that may have been adaptive (from an evolutionary perspective) after severe peripheral injury but is maladaptive after SCI. Evidence that hyperfunctional nociceptors make large contributions to behavioral hypersensitivity after SCI suggests that nociceptor-specific ion channels required for nociceptor SA and hypersensitivity offer promising targets for treating chronic pain and hyperreflexia after SCI.

## Introduction

Neuropathic pain in general and spinal cord injury (SCI) pain in particular are usually viewed as maladaptive consequences of neural injury (Costigan et al., 2009; Yezierski, 2009; Gwak and Hulsebosch, 2011). Chronic neuropathic pain is certainly maladaptive for patients because of the suffering and disability that can occur. A broader view is that SCI results in the pathological recruitment of mechanisms that under other conditions can be biologically adaptive. I will argue that SCI presents a complex set of signals to primary nociceptors that induces a persistent hyperfunctional state in these neurons that can be triggered by extrinsic *central signals* that are associated with severe but survivable *peripheral injury*. My focus is on the many uninjured nociceptors that are chronically altered by SCI and positioned to help drive below-level and at-level pain.

## Central neuropathic pain involves many mechanisms, some of which may involve enhanced activity in primary afferent neurons

Before considering the role of primary nociceptors in driving chronic SCI pain, I note that numerous mechanisms at many different central loci may contribute to central neuropathic pain, and different forms of central pain are likely to differ in their dependence upon alterations in primary sensory neurons. Injury to any part of a pain pathway could result in chronic pain if the injury persistently enhances excitatory activity within the pathway. For example, small strokes in the thalamus and other parts of the brain can produce chronic pain (Finnerup, 2008), and these injuries and their sequelae may have little impact on primary afferent neuron function.

Some injuries to the spinal cord might also produce chronic pain with relatively little involvement of primary afferent neurons. For example, a small electrolytic lesion encompassing part of the spinothalamic tract in the cervical spinal cord causes persistent behavioral hypersensitivity (Masri et al., 2009) and tonic pain (Davoody et al., 2011), possibly by disinhibition of thalamic pathways (Masri et al., 2009). More extensive spinal injuries that are more likely to impact primary afferents produce many central alterations that, in principle, can underlie chronic pain. For example, SCI causes disinhibitory effects within the dorsal horn by severing descending inhibitory pathways (Bruce et al., 2002; You et al., 2008), killing inhibitory interneurons (Meisner et al., 2010), reducing spinal GABA levels (Gwak et al., 2008), and changing the Cl^−^ equilibrium potential in projection neurons receiving inhibitory input (Lu et al., 2008). In addition, long-lasting intrinsic hyperexcitability of central neurons within pain pathways can occur after SCI. Notably, hyperexcitability is expected from the upregulation of Na^+^ channels described in spinal dorsal horn neurons and thalamic neurons (Hains et al., 2003, 2005). There is also morphological evidence for synaptic potentiation in dorsal horn neurons after SCI (Tan and Waxman, 2012). Finally, SCI causes long-lasting alterations in spinal microglia and astroglia that are reported to promote pain-related behavior (e.g., Hains and Waxman, 2006; Zhao et al., 2007; Detloff et al., 2008; Carlton et al., 2009; Marchand et al., 2009; Tan et al., 2009; Gwak et al., 2012).

Finding long-lasting alterations in central pain pathways after SCI does not demonstrate that alterations within the CNS are exclusively responsible for chronic pain. Central alterations might be driven in large part by chronic increases in the activity of primary sensory neurons. Primary afferents are clearly capable of driving chronic pain; for example, their persistent activity plays a major role in maintaining pain in some peripheral neuropathy models (e.g., Gracely et al., 1992; Zhang et al., 2000; Sukhotinsky et al., 2004; Xie et al., 2005; Pitcher and Henry, 2008).

## SCI alters central and peripheral branches of primary nociceptors

The first evidence for SCI-induced alterations of primary sensory neurons came from observations suggesting that primary afferents, and especially those immunoreactive for CGRP (a marker for “peptidergic nociceptors”), sprout new branches within the dorsal horn after SCI, which potentially could lead to more extensive nociceptive input to dorsal horn neurons (Helgren and Goldberger, 1993; Christensen and Hulsebosch, 1997; Krenz and Weaver, 1998; Weaver et al., 2001; Ondarza et al., 2003; Zinck et al., 2007; Hou et al., 2009). Although a failure to find evidence of sprouting of CGRP-positive fibers has been reported in some SCI models (Kalous et al., 2007, 2009), the possibility of nociceptor sprouting after spinal contusion injury is supported by the finding that SCI triggers an intrinsic growth-promoting state that further enhances the growth in culture of dissociated small and medium-sized DRG neurons, including CGRP-positive neurons, sampled from spinal segments close to and distant from an injured segment (Bedi et al., 2012).

Physiological evidence for persistent hyperexcitability of C-fiber neurons after SCI first came from observations of increases in TTX-sensitive Na^+^ currents in dissociated somata of primary afferents innervating the bladder (Yoshimura and de Groat, 1997; de Groat and Yoshimura, 2010). An association of persistent hyperexcitability in nociceptors with pain-related behavior was not reported until twelve years later. A contusion injury at T10 that produced mechanical and thermal hypersensitivity of forelimb withdrawal responses tested 35 days later increased the sensitivity of the peripheral terminals of functionally identified nociceptors to mechanical and thermal stimulation in an isolated forepaw skin-nerve preparation, as well as producing low-frequency spontaneous activity (SA) in the terminals of the nociceptors (Carlton et al., 2009). This important discovery suggested that hyperexcitability and SA in peripheral branches of primary nociceptors might help to drive chronic pain after SCI, although it did not show whether this hyperexcitability was an intrinsic property of the nociceptors.

## SCI induces persistent spontaneous activity (SA) in nociceptor somata

SCI has surprisingly strong effects on the excitability of nociceptor somata (cell bodies) (Bedi et al., 2010). *In vivo* recordings from nociceptive C and Aδ fibers in anesthetized rats showed that contusive SCI dramatically increased the incidence of SA generated within L4 and L5 DRG 1 to 3 months after injury. SCI-induced SA in nociceptor somata was also expressed *in vitro*, 1 day after dissociation in low-density cultures lacking serum or growth factors. The incidence of *in vitro* SA was remarkably high (35–70%) 3 days to 8 months after SCI compared to what has been reported (0–20%) for dissociated small DRG neurons sampled from rats with peripheral neuropathy (Petersen et al., 1996; Abdulla and Smith, 2001; Ma and LaMotte, 2005; Zheng et al., 2007). All of the spontaneously active neurons sampled *in vitro* after SCI were small, ~80% were capsaicin sensitive, and ~33% bound isolectin B4 (a positive marker for “non-peptidergic” nociceptors; Wang et al., 1994), indicating that most of the neurons exhibiting SA were nociceptors (Bedi et al., 2010). The incidence of SA after SCI was highest in neurons dissociated from DRG far below the injury level (L4/L5, 50–70%) and high just below the injury (T11/T12, 35–50%) compared to the incidence in dissociated neurons from sham-operated animals (16%) and naive animals (15%). Somally generated SA was not elevated significantly in neurons from DRG far above the injury (C6/C7, 10–25%), even though SCI enhanced peripherally generated SA in nociceptors at this level (Carlton et al., 2009). Another difference between nociceptor effects above and below the injury level was the absence of elevated SA just above the injury (T8/T9, 10%) 3 days after SCI, whereas 1 month or later 50% of T8/T9 neurons exhibited SA. Thus, contusive SCI can persistently increase the prevalence of somally generated SA close to and far below the injury site. It is not yet known if peripherally generated SA occurs in nociceptors outside the cervical region. Finding the highest incidence of SA in small, C-type neurons distant from the injury site shows that axotomy of these neurons by SCI is not required to trigger the chronic SA (see below). Enhancement of the incidence of nociceptor SA by SCI is not restricted to spinal contusion injury; in preliminary studies 70% of below-level DRG neurons dissociated 1–3 months after spinal hemisection injury at T10 showed SA (Carlton et al., 2011), and 80% showed SA 1 month after unilateral avulsion of T13/L1 dorsal roots (Du et al., 2011). In both cases there was similar incidence of SA in dissociated neurons from DRG ipsilateral and contralateral to the injury, which again shows that direct injury of nociceptor axons is not an important trigger for chronic SA after SCI. Note that unilateral dorsal root avulsion, like most forms of SCI, causes chronic pain (Wieseler et al., 2010) and widespread, bilateral inflammatory and glial responses in the spinal cord (Chew et al., 2011).

## SCI induces a discrete hyperexcitable/spontaneously active (HSA) state in nociceptor somata

Differences in various somal properties related to hyperexcitability were found in nociceptors dissociated from SCI animals compared to those from sham-operated and naïve animals (Bedi et al., 2010). Surprisingly, all the observed differences in excitability properties could be accounted for by the existence of a single population of hyperexcitable/SA (HSA) nociceptors, which was large in SCI animals and much smaller in naive and sham-treated animals. Compared to silent neurons, SA neurons from any of the treatment groups were depolarized at rest by nearly 5 mV, exhibited more repetitive firing during depolarizing test pulses, required less depolarizing current to evoke action potentials, and had much lower membrane conductance when tested within the range of membrane potentials where SA occurred. The stronger correlation among all the hyperexcitability properties and SA than between any of these properties and SCI indicates that SCI promotes entry of nociceptor somata into a discrete HSA state. The expression of the HSA state 1 day after dissociation and 5 or more months after SCI shows that the intrinsic HSA state is persistent. The persistence could come from a *stable HSA state* that, once induced, is expressed for very long periods in similar fashion *in vivo* and *in vitro*, even in the absence of inflammatory signals and growth factors. This possibility is supported by the high incidence of SA recorded in dissociated DRG neurons and *in vivo* from axons in dorsal roots connected to the DRG but disconnected from the periphery and spinal cord, and the similarity of the spontaneous firing rates (~1 Hz) under these quite different conditions (Bedi et al., 2010). Persistence could also come from a *stable hypersensitive state* in which a nociceptor's predisposition to enter a more transient HSA mode is enhanced, but remains latent unless inflammatory mediators or other injury-related stimuli are present. Such stimuli might include signals generated during the injurious process of dissociation, which by itself can modestly promote entry into an HSA-like state, increasing the incidence of nociceptor SA from ~0 to ~13% (Zheng et al., 2007)—much lower than the >50% incidence of SA in dissociated nociceptors sampled from DRG after SCI (Bedi et al., 2010). A very long-term, SCI-induced intrinsic hypersensitive state that enhances a nociceptor's responding with an intermediate-term HSA mode to inflammation- and injury-related signals would be consistent with the phenomenon of “hyperalgesic priming” that has been extensively studied in nociceptors (Reichling and Levine, 2009) and is discussed below.

## SA in nociceptor somata is correlated with and may help drive pain-related behavior after SCI

The incidence of somal SA after SCI was significantly correlated with behavioral hypersensitivity tested 1 and 3–5 months after injury; the animals showing the greatest sensitivity to mechanical and thermal test stimuli applied to all four paws also had the highest incidence of nociceptor SA recorded *in vitro* (Bedi et al., 2010). Significant correlations between mechanical or thermal hypersensitivity and incidence of nociceptor SA were found for hindlimb responses, which were correlated with SA in neurons from L4/L5 DRG. Furthermore, forelimb responses were correlated with SA in the above-level neurons sampled from T8, T9, C6, and C7 DRG. Particularly interesting effects of SCI were found on vocalization elicited by mechanical stimuli delivered to an array of test sites on the back. SCI dramatically reduced the incidence of vocalization evoked by below-level test stimuli, suggesting substantial interruption of spinal pathways traversing the injury site. Conversely, SCI increased the incidence of vocalization to above-level stimuli, and the above-level vocalization was correlated with SA in neurons sampled from at- and above-level DRG. Surprisingly, relatively little chronic SA was observed in somata of neurons from C6/C7 DRG; influences of nociceptor SA on supraspinal responses and forelimb responsiveness may come from wide-ranging effects of active nociceptors in above-level DRG closer to the injury site, or from nociceptor SA generated in the periphery (Carlton et al., 2009).

SA generated within somata and peripheral branches of nociceptors is likely to drive central sensitization (Carlton et al., 2009; Bedi et al., 2010). If this SA also drives pain-related behavioral alterations, then manipulations that selectively block the SA should reduce the behavior. The nociceptor-specific Na^+^ channel, Nav1.8 is important for the expression of nociceptor SA and other sensitizing effects in other pain models (Lai et al., 2002; Roza et al., 2003; Jarvis et al., 2007; Abrahamsen et al., 2008). Importantly, knocking down the expression of Nav1.8 largely eliminates SCI-induced nociceptor SA *in vitro* and greatly reduces behavioral hypersensitivity to mechanical and thermal test stimulation applied *in vivo* (Yang et al., 2012). This finding indicates that SA and hyperexcitability in primary nociceptors plays a major part in driving chronic hypersensitivity and possibly pain after SCI.

## Persistent nociceptor alterations are hypothesized to be triggered by somal integration of central and local information that indicates severe injury

**Figure 1:**
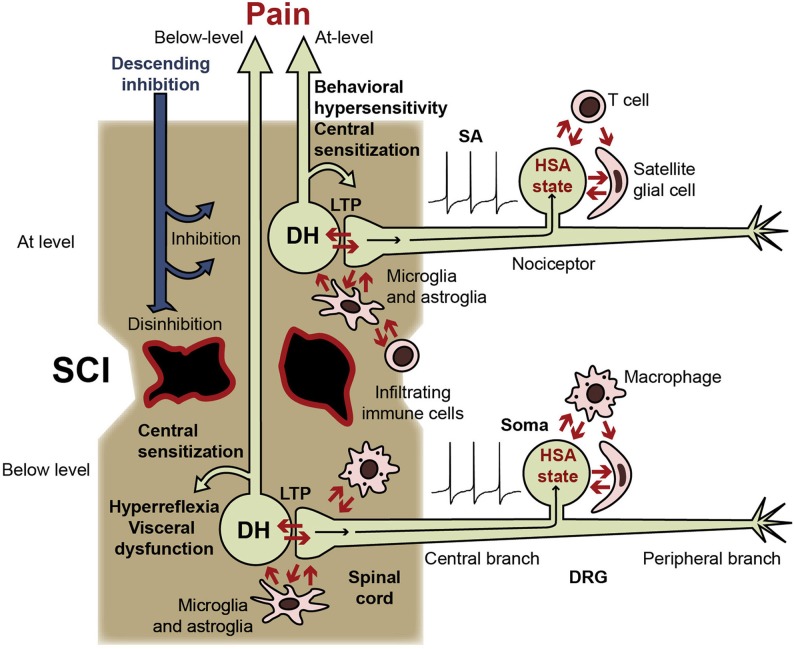
**Central and somal signals received by nociceptors during SCI, and consequences of switching nociceptors into a persistent hyperfunctional state.** Nociceptors receive injury-related signals within the spinal cord (from intensely activated postsynaptic dorsal horn neurons [DH], activated glia, and infiltrating immune cells) and within the DRG (from other DRG neurons, satellite glial cells, and the blood). Nociceptors have potent excitatory effects on pain pathways (indicated by DH neurons) and on circuits subserving somatic and visceral functions (not shown). LTP at DH synapses can be produced by somal and peripheral SA and afterdischarge, facilitated by the nociceptor hyperfunctional state (including the somal hyperexcitable/SA [HSA] state). Nociceptor activity produces central sensitization, promotes spontaneous and evoked pain, and enhances somatic and visceral reflexes. Nociceptor activity also leads to positive feedback interactions with postsynaptic neurons, other DRG somata, inflammatory cells (microglia, infiltrating macrophages, and T cells), astrocytes, and satellite glial cells. Similar interactions of peripheral branches with surrounding cells are possible, but are less likely after central than peripheral injury and are not indicated here. Because SCI severs or demyelinates many ascending fibers, much of the activity in pain pathways generated below the injury level may be blocked, although residual pathways (illustrated) are likely to contribute to the sensation of below-level pain in cases of incomplete SCI. Conversely, interruption of descending inhibitory pathways enhances spinal excitability, promoting entry of nociceptors into the hyperfunctional state and further increasing the somatic and autonomic hyperreflexia and visceral dysfunction driven by SA in below-level nociceptors. Nociceptor SA generated immediately above the injury level should have ready access to intact spinal circuits and projection neurons in pain pathways, contributing to central sensitization, behavioral hypersensitivity, and at-level pain. At-level nociceptor alterations may involve additional signals generated by direct damage to the nociceptor (axotomy) and to nearby cells.

My central hypothesis is that a long-lasting hyperfunctional state (which includes the somal HSA state) can be triggered in nociceptors by a combination of events after SCI that mimic patterns of signals used by nociceptors to recognize particularly severe *peripheral* injury. In the next sections I discuss the types of signals that this hypothesis suggests are integrated by the nociceptor soma to induce and maintain a persistent hyperfunctional state (Figure 1). I then present adaptive arguments for this hypothesis, and discuss the nature of the hyperfunctional state and some of its pathological consequences after SCI.

Long-lasting changes in intrinsic functional properties of primary sensory neurons are likely to depend upon changes in gene expression in these neurons (e.g., Lewin and Walters, 1999; Woolf and Costigan, 1999; Thompson et al., 2004; Lee et al., 2008; von Schack et al., 2011), although continuing exposure to extrinsic signals might also maintain functional changes (see below). Assuming that changes in nociceptor gene transcription are necessary for a persistent hyperfunctional state, what types of information does a nociceptor nucleus use to decide whether to enter this state?

One potential set of signals that will *not* be emphasized here is produced by direct injury (axotomy) to the nociceptor's own branches. This includes immediate injury discharge and both positive injury signals (e.g., activated protein kinases), and negative injury signals (e.g., a decrease in target-derived growth factors) transported from damaged axons to the nucleus (Ambron and Walters, 1996). Persistent functional changes in primary afferent neurons associated with changes in gene expression are prominent in axotomizing peripheral nerve injury models (e.g., Obata et al., 2003; Persson et al., 2009). Axotomy-induced and demyelination-dependent signals, especially in nociceptors and other primary afferent neurons close to the injury level, may also contribute to chronic pain after SCI, as may other effects at the injury site, such as glial scarring. The constellation of injury signals expressed solely at the spinal injury level is likely to contribute to at-level pain and may distinguish SCI pain from other forms of chronic pain. These at-level injurious effects may not contribute directly to below-level or above-level pain.

In contrast, the present hypothesis focuses on signals causing alterations in nociceptors that have *not* been injured. SA and hyperexcitability or sensitization are prominent in nociceptors distant from a T10 contusion site (in neurons from lumbar and cervical DRG), which are unlikely to have axons projecting close enough to the lesion to experience axotomy or other forms of direct damage (Carlton et al., 2009; Bedi et al., 2010; see also Chung et al., 1979; Traub et al., 1990; Huang et al., 2006). Furthermore, uninjured nociceptors after SCI (Bedi et al., 2010) display much higher incidence of SA than reported for peripherally axotomized nociceptors (Liu et al., 2000; Djouhri et al., 2006). A functional consideration is that signals of injury to a nociceptor's own branches (axotomy signals) provide little information to the soma about the severity of a bodily injury because the natural signal source is restricted to a single, small receptive field. As outlined in the following sections, I propose that nociceptors assess the severity of peripheral injury by integrating nociceptive information coming from the spinal cord, other cells within the DRG, and blood, and that SCI happens to produce similar combinations of signals, leading to a maladaptive induction of the nociceptor hyperfunctional state.

## Central inflammatory signals are hypothesized to provide injury-related information to nociceptors

In the periphery, prolonged or repeated inflammation in the absence of axotomy often causes generation of nociceptor SA near a site of inflammation in peripheral terminals (Djouhri et al., 2006; Xiao and Bennett, 2007), along uninjured axons adjacent to degenerating axons (Campbell, 2001; Wu et al., 2001), and within the DRG (Xie et al., 2006; Huang et al., 2011). Inflammation makes major contributions to chronic pain in some peripheral neuropathy models that lack extensive axotomy (e.g., Clatworthy et al., 1995; Miller et al., 2009; Bastos et al., 2012). Importantly, peripheral injury or inflammation produces central inflammatory effects, including pronounced activation of microglia in the dorsal horn (e.g., Fu et al., 1999; Xie et al., 2009; Kim and Moalem-Taylor, 2011). After SCI (Figure 1), the central branches of nociceptors are exposed for long periods to inflammatory mediators released by resident microglia and infiltrating immune cells (Alexander and Popovich, 2009; Byrnes et al., 2011), as well as cytokines and chemokines released from astroglia and even from nociceptors themselves (see Miller et al., 2009). Enhanced expression of cytokines is found near the injury site and also in distant spinal segments months after SCI (Detloff et al., 2008; Hulsebosch, 2008; Sandhir et al., 2011). Although little is yet known about effects of central inflammation on nociceptors, peripheral inflammation or treatment with inflammatory mediators causes an upregulation in DRG neurons of numerous molecules that can increase nociceptor excitability, including TRPV1 (Ji et al., 2002; Yu et al., 2008), TRPA1 (Katsura et al., 2006), Nav1.7 (Strickland et al., 2008), and Nav1.8 (Coggeshall et al., 2004; Villarreal et al., 2005; Strickland et al., 2008). In addition, inflammation can downregulate K^+^ channels (La and Gebhart, 2011; Marsh et al., 2012). Whereas alterations of axotomized nociceptors after nerve injury involve reduced access to target-derived trophic factors, which can result in conflicting effects on nociceptive function (e.g., decreased expression of both Na^+^ and K^+^ channels) (Costigan et al., 2009), alterations of uninjured nociceptors after inflammation typically result in increased levels of trophic factors and other inflammatory mediators, enhancing nociceptive function (e.g., by increasing activity and expression of Na^+^ channels and decreasing activity and expression of K^+^ channels) (Gold and Gebhart, 2010; Linley et al., 2010; Marsh et al., 2012). Although little is known about the molecular alterations underlying SCI-induced hyperexcitability and SA in nociceptors, the effects observed thus far resemble those accompanying persistent peripheral inflammation: increased sensitivity to capsaicin and upregulation of TRPV1 channels (Wu et al., 2011), and a dependence upon Nav1.8 channels (Yang et al., 2012), suggesting that the hyperfunctional state depends upon prolonged exposure of a nociceptor's central terminals (and perhaps its soma) to inflammatory signals.

## Retrograde signals from intensely activated postsynaptic neurons in the dorsal horn are hypothesized to provide injury-related information to nociceptors

Summation within dorsal horn neurons of the synaptic effects of numerous nociceptors activated by severe peripheral injury and/or inflammation will lead to synaptic LTP, which is likely to be accompanied by the generation of a series of retrograde synaptic and axonal signals that are transported back to the nucleus of presynaptic nociceptors (Parada et al., 2003; Perry and Fainzilber, 2009; Ho et al., 2011). As one example of several potential retrograde synaptic signals that might also be activated after SCI, bidirectional ephrinB-EphB signaling between nociceptors and dorsal horn neurons promotes long-lasting behavioral hypersensitivity, LTP, and upregulation of ephrinB1 in nociceptor somata after peripheral nerve injury (Song et al., 2008a,b). After SCI (Figure 1) intense activation of postsynaptic neurons in the dorsal horn will be produced by direct excitation from active nociceptors and from activated microglia and astroglia, and will be amplified by interruption of descending neural inhibition (Hains and Waxman, 2006; Hulsebosch, 2008; Carlton et al., 2009; Marchand et al., 2009; Miller et al., 2009; Gwak et al., 2012). Signaling from neurons and glia might also lead to initiation of action potentials within the central terminals of nociceptors (Lin et al., 2000; Price et al., 2009) that could signal retrogradely to the soma. Interestingly, continuing LTP of nociceptor synapses might also be a direct consequence of SA in these cells. C-fiber LTP *in vivo* occurs at very low firing frequencies (e.g., 2 Hz) (Drdla and Sandkuhler, 2008), well within the range of SA firing rates observed after SCI (Bedi et al., 2010). SA-driven LTP and retrograde signals arising during LTP might contribute to positive feedback interactions between nociceptors and postsynaptic targets after SCI.

## Extracellular chemical signals within the DRG are hypothesized to provide injury-related information to nociceptors

Extracellular signals (in addition to the axonally transported intracellular signals just described) are conveyed directly to cells within the DRG after peripheral injury/inflammation and after SCI (Figure 1). Peripheral injury and inflammation will activate numerous nociceptors and this electrical activity will lead to release of neurotransmitters, including neuropeptides and chemokines (Miller et al., 2009), not only from nociceptor terminals in the dorsal horn, where they can activate neurons and glia (Wen et al., 2007), but also from the somata of active nociceptors within the DRG (Huang and Neher, 1996; Zhang and Zhou, 2002; Zhang et al., 2007; Jung et al., 2008), leading to the stimulation of satellite glial cells (Zhang et al., 2007), and probably other DRG neurons (Devor and Wall, 1990). Peripheral nerve injury can lead to the infiltration of hematogenous immune cells into the DRG (Kim and Moalem-Taylor, 2011) and spinal cord (Grace et al., 2011). Similarly, spinal transection causes infiltration of macrophages and T cells into DRG close to and distant from the injury site (McKay and McLachlan, 2004), where they might stimulate nociceptor somata. Severe peripheral or central injury will also cause the release of numerous extracellular signaling molecules into the blood. For example, SCI causes systemic inflammation (Gris et al., 2008) and long-term elevation of circulating cytokines (Davies et al., 2007). Because the DRG, unlike the CNS or nerves, lacks an effective vascular permeability barrier (Abram et al., 2006; Jimenez-Andrade et al., 2008), nociceptor somata and satellite glial cells will be fully exposed to systemic, blood-borne signals of injury, and inflammation.

## Several mechanisms are hypothesized to maintain the nociceptor hyperfunctional state

Three general possibilities exist for maintaining the hyperfunctional state: (1) continuing release of extrinsic signals, such as inflammatory mediators, that continuously refresh the hyperfunctional state, (2) positive feedback loops between nociceptor activity and inflammatory and retrograde synaptic effects, and (3) switching of the nociceptor into a potentially permanent intrinsic hyperfunctional state that remains after the extrinsic induction signals fade (“nociceptor memory”). First, widespread inflammation may persist chronically after SCI (Byrnes et al., 2011; Pajoohesh-Ganji and Byrnes, 2011). Moreover, macrophages and microglia can show priming that may outlast the apparent resolution of inflammation for weeks (Hains et al., 2010), suggesting a form of cellular memory in these inflammatory cells. Second, because activity in nociceptors leads to the release or activation of signals from interacting cells (postsynaptic neurons, glia, immune cells, neighboring DRG neurons, and peripheral cells) that can stimulate the nociceptors, ongoing positive feedback between nociceptors and various target cells may contribute to the persistence of the nociceptor hyperfunctional state (Miller et al., 2009; Xie et al., 2009). Third, cellular memory within nociceptors may be particularly important. The SCI-dependent somal HSA state was found to persist for at least 1 day after isolation of nociceptors (Bedi et al., 2010), but the full duration of this intrinsic state is unknown. Peripheral models of chronic inflammatory pain show that nociceptor alterations can persist for weeks in the absence of obvious continuing inflammation. Notably, hyperalgesic priming is produced by a single episode of acute cutaneous inflammation in a rat hindpaw (e.g., from carrageenan injection) or brief injection of an inflammatory signal (e.g., TNFα, NGF, and GDNF), which is followed days or weeks after abatement of the resulting acute pain by dramatically increased sensitivity of nociceptors to a subsequent inflammatory challenge (usually injection of PGE_2_) (e.g., Aley et al., 2000; Reichling and Levine, 2009; Alvarez et al., 2012; Bogen et al., 2012). Moreover, repeated daily injections of PGE_2_ into a hindpaw under a condition (concurrent indomethacin application) that avoids apparent tissue inflammation produce behavioral hypersensitivity for at least 1 month afterwards, which is accompanied by Nav1.8 upregulation and is dependent upon Nav1.8 expression, suggesting a key role for intrinsically altered nociceptors (Villarreal et al., 2005). A number of memory-like modifications involving hyperexcitability and synaptic facilitation have been described in various nociceptors (e.g., Walters et al., 1991; Kandel, 2001; Weragoda et al., 2004; Bogen et al., 2012; Zhang et al., 2012) and some of these modifications might be triggered by signals associated with SCI.

## SCI is hypothesized to cause maladaptive activation of a nociceptor hyperfunctional state that may be biologically adaptive after severe peripheral but not central injury

**Figure 2:**
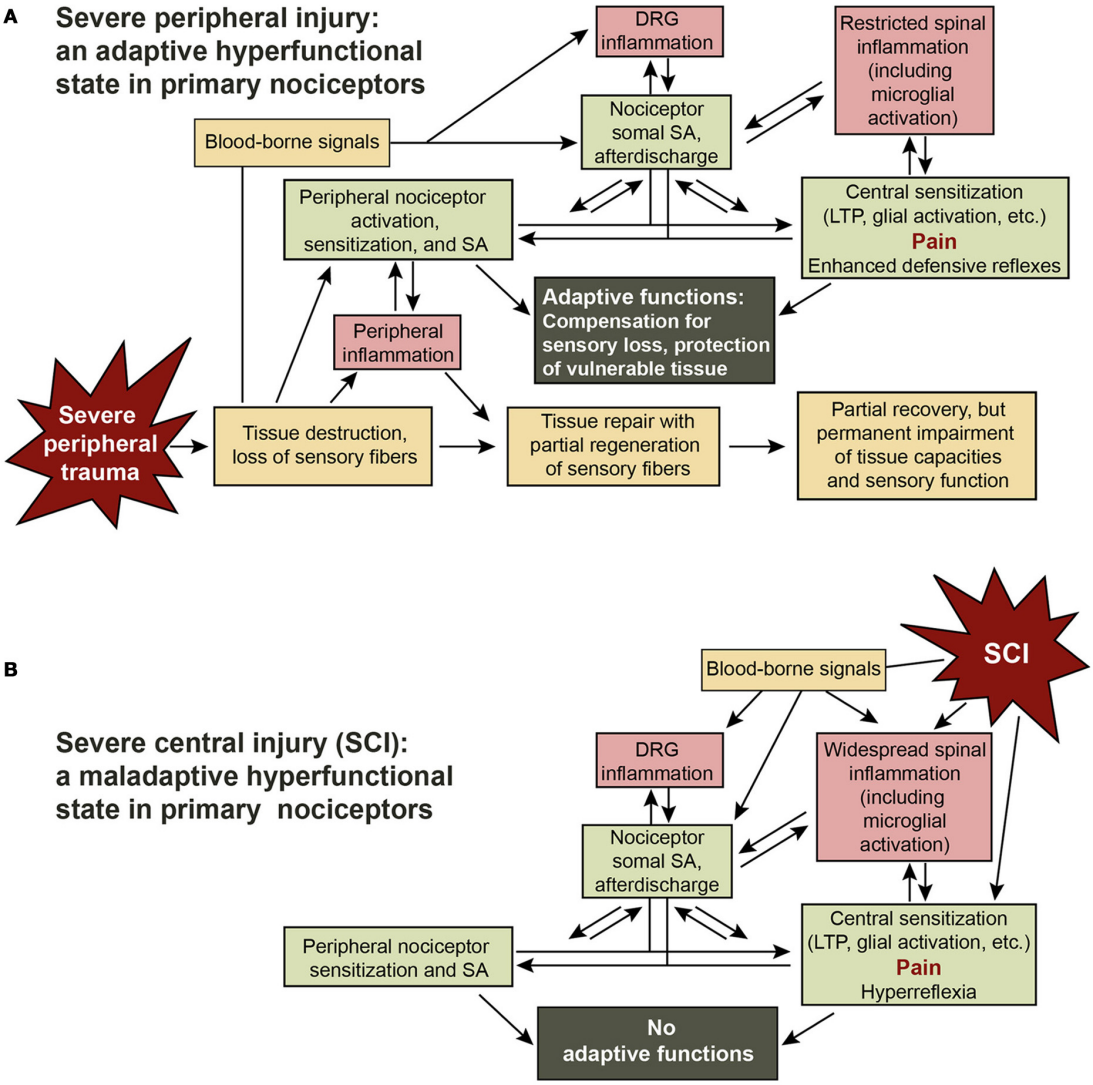
**Adaptive-maladaptive nociceptor hyperfunctional state hypothesis. (A)** Adaptive nociceptor-driven sensitization and pain after severe peripheral injury. Compensation for permanent impairment of peripheral sensory function and protection of weakened tissue are achieved by enhancing the function of surviving nociceptors that innervate the region of injury via peripheral sensitization and by peripheral and somal hyperexcitability that produce SA, afterdischarge, LTP, and activation of central neurons and glia. Some degree of localized chronic pain can be useful for enhancing awareness of and protecting a chronically weakened body part. The decision to enter a persistent hyperfunctional state requires integration by the nociceptor soma of injury-related information from the peripheral receptive field, other cells in the DRG, cells in the spinal cord, and signals in the blood. **(B)** Maladaptive triggering of the nociceptor hyperfunctional state by SCI. SCI leads to the generation of many of the same signals in the spinal cord, DRG, and blood that are produced during severe peripheral injury, switching numerous nociceptors into a persistent hyperfunctional state. In this case the consequent sensitization, SA, hyperreflexia, and pain have no adaptive functions.

My central hypothesis is that severe peripheral injury (Figure 2A) generates a complex set of central signals that triggers a biologically adaptive and highly persistent nociceptor hyperfunctional state, and that many of these signals are also generated by SCI, resulting in maladaptive chronic pain and hyperreflexia in SCI patients (Figure 2B). It is evident that chronic pain, like other clinical consequences of SCI, is maladaptive for patients. Furthermore, a long-lasting hyperfunctional state induced by SCI should not be adaptive in the evolutionary sense of increasing survival and reproductive success because SCI, like all major CNS trauma, is almost always fatal to mammals in the absence of medical intervention (Branco et al., 2007; Weil et al., 2008). Any adaptiveness of this state must be for conditions other than SCI—although SCI does provide an extremely favorable setting for pathological recruitment of the nociceptor hyperfunctional state (see below). The proposal that human SCI pain involves the maladaptive recruitment of a nociceptor state that has adaptive functions under other conditions may seem questionable for at least two reasons. First, the SCI-induced HSA state found in isolated nociceptor somata might be a purely pathological effect, perhaps amplified by the abnormal conditions of dissociated cell culture (Zheng et al., 2007). However, the finding of SA generated within DRG *in vivo* months after SCI (Bedi et al., 2010) suggests that the nociceptor hyperfunctional state is not an artifact of cell culture. Second, it has not yet been established in mammals that severe peripheral injury induces a chronic hyperfunctional state in nociceptors, or that such a state is adaptive in that context. These assumptions are supported by the following arguments.

A shared function of nociceptors and the innate immune system, which mediates most of the inflammatory responses to trauma, is to protect the body during and after survivable injury. This function is served by bidirectional communication between nociceptors and the innate immune system, including granulocytes and macrophages in the periphery, and resident microglia in the spinal cord (Miller et al., 2009). The adaptive immune system also appears to play a role after peripheral injury, with T cells crossing the blood-spinal cord barrier and promoting pain by interactions with microglia (Grace et al., 2011). More severe injuries are associated with greater inflammation and longer periods of repair and recovery. Whereas severe CNS injuries are fatal in the absence of medical care, many animals can sometimes survive quite severe peripheral injuries, especially to appendages, without medical treatment. Examples include accounts of wolves surviving after loss of a limb in a trap, and humans surviving the amputation of digits or even limbs without medical care. A common consequence of severe peripheral injury is incomplete recovery of tissue function (Figure 2A), especially if muscle, bone, and nerves are seriously damaged. Resulting sensory loss can be a life-threatening problem because damaged tissue is especially vulnerable, both to inadvertent self-inflicted injury during movement and to attacks from predators and parasites that are attracted to signs of bodily injury (Walters, 1994).

Sensitization to mechanical and other types of stimuli, as well as additional hyperfunctional changes in surviving sensory fibers in and around injured tissue can both compensate for loss of sensory function and help protect the vulnerable region by promoting protective behavior (Walters, 1994; Smith and Lewin, 2009). Hyperfunctional alterations in nociceptors include peripheral sensitization and hyperexcitability (Gold and Gebhart, 2010), with the latter expressed not only as lower action potential threshold but as afterdischarge (Clatworthy and Walters, 1993; Gasull et al., 2005; Weng et al., 2012) and SA. Sensory function can also be enhanced by increasing the synaptic effectiveness of surviving nociceptors, which occurs after peripheral inflammation and nerve injury (Walters et al., 1991; Woolf and Costigan, 1999; Ikeda et al., 2006; Song et al., 2008a,b; Zhao et al., 2010), and is likely after SCI (Tan and Waxman, 2012). These effects should be coordinated with restoration of sensory function by regenerative and compensatory growth (sprouting) in peripheral and potentially central compartments of the nociceptor (Kinnman and Aldskogius, 1986; Doucette and Diamond, 1987; Billy and Walters, 1989; Steffensen et al., 1995; Belyantseva and Lewin, 1999; Hill et al., 2010; Bedi et al., 2012). Hyperfunctional alterations might, in addition, include enhanced sensitivity of nociceptors to chemical signals associated with injury (Song et al., 2003b), such as endogenous activators of TRPV1 channels (Patapoutian et al., 2009; Patwardhan et al., 2009; Diogenes et al., 2011; Wu et al., 2011). Traumatic injuries accompanied by extensive tissue disruption are likely to interrupt pathways that permit successful sensory regeneration or compensatory sprouting, so that substantial amounts of tissue may remain deprived of sensory innervation after tissue repairs are completed and inflammation subsides. In such cases it could be adaptive for compensatory alterations to persist for very long periods, perhaps for the remainder of the injured animal's reproductive life. Indeed, selection for persistent compensatory alterations in phylogenetically ancient sensory neurons after severe peripheral injury has been suggested as an early stage in the evolution of mechanisms that later found uses in some forms of long-term memory (Walters and Moroz, 2009).

Hyperfunctional alterations in low-threshold primary afferent neurons that detect innocuous stimuli and can elicit rapid defensive responses to potentially threatening but not tissue-damaging stimuli could also be adaptive after severe peripheral trauma. Even though Aβ-type neurons exhibit SA and other alterations in some chronic pain models (e.g., Gracely et al., 1992; Devor, 2009; Song et al., 2003a, 2006, 2012; Xie et al., 2012), it is not yet known if low-threshold mechanosensory neurons express a persistent, intrinsic hyperfunctional state after injury or inflammation.

## The nociceptor soma can be a site for the generation of adaptive electrical activity

In principle, somal as well as peripheral and synaptic alterations in nociceptors are likely to help compensate for loss of peripheral sensory branches after severe peripheral injury (and to contribute to pain after SCI, and perhaps to phantom pains after amputation). Somata of nociceptors identified thus far in vertebrates, molluscs, and annelids are often located centrally, distant from their peripheral receptive fields, which ensures that these cells can survive sublethal injury that destroys their peripheral branches. In the mollusc, *Aplysia*, peripheral injury induces somal hyperexcitability lasting months that amplifies brief bursts of action potentials arriving from the periphery by generating high-frequency afterdischarge (Clatworthy and Walters, 1993; Gasull et al., 2005), and similar afterdischarge has been described in the somata of mammalian Aβ afferent neurons (Song et al., 2012). This possibility has not yet been tested in the somata of vertebrate nociceptors, although nociceptor afterdischarge is generated peripherally (Weng et al., 2012). SA and enhanced discharge generated anywhere within a nociceptor would be expected to sensitize defensive responses, and could also stimulate conscious, protective attention to the injured region (pain). In the case of nociceptors that have lost peripheral branches in an injured region, somally generated SA could achieve this result. Moreover, even in nociceptors with surviving peripheral branches, somally generated SA and afterdischarge may reach the CNS more reliably than activity generated peripherally. Under normal conditions nociceptor action potentials are subject to significant conduction block where distal branches join the main axon (Sun et al., 2012), and this conduction block should be greatly enhanced by inflammatory conditions (e.g., via inactivating depolarization of axons) and by structural changes associated with tissue disruption and the small diameters of regenerating fibers. Thus, somally generated SA in a nociceptor innervating a severely injured region may be an effective way to ensure maintained sensitization of appropriate defensive responses and awareness of a vulnerable part of the body that requires extra attention (Figure 2A) even if regeneration of injured sensory fibers is limited.

Why haven't chronic, somally expressed HSA states in nociceptors been reported previously in mammalian models of long-lasting pain? Few chronic studies have tested somal excitability in nociceptors, or recorded under conditions where nociceptor SA generated in the DRG could be distinguished from SA generated peripherally. Moreover, most studies of dissociated nociceptors have examined ionic currents under voltage clamp, without testing for somal hyperexcitability or SA. In addition, common models of long-lasting pain, including models based on cutaneous injection of inflammogens, surgical incision models (which produce relatively little inflammation or nerve damage), and various nerve injury models may not mimic adequately severe peripheral injuries that involve extensive amounts of tissue destruction (including neural damage) combined with prolonged inflammation—i.e., the traumatic conditions most likely to result in chronic pain in humans. Thus, while standard pain models produce many sensitizing effects on nociceptors (Gold and Gebhart, 2010), these pain models may not be severe enough to induce the persistent somal HSA state in sufficient numbers of nociceptors for it to be detected readily.

## Nociceptor SA may be a useful target for treating pain and hyperreflexia after SCI

Nociceptor activity has extremely powerful effects on sensation, behavior, emotion, and autonomic function, so chronic activity and hyperexcitability occurring in numerous nociceptors after SCI would be expected to be clinically significant (Figure 2B). Chronic SCI pain in humans can be felt in many parts of the body, but is most commonly experienced in segments near the injury (at-level pain) and below the injury (below-level pain) (Siddall et al., 2003; Finnerup and Jensen, 2004). Spontaneous and evoked activity in below-level nociceptors (Figure 1) should help excite and sensitize local circuits mediating segmental responses such as hindlimb withdrawal reflexes, and help drive below-level spontaneous pain, allodynia, and hyperalgesia. By definition, pain includes an emotional, subjective component, which in mammals involves cortical processing (e.g., Melzack, 2005; Neugebauer et al., 2009). Thus, below-level pain caused by below-level nociceptor activity would require that a sufficient number of ascending fibers remain functional (intact and still myelinated), and this probably occurs in many incomplete spinal injuries (Wasner et al., 2008; Densmore et al., 2010). However, observations that moderate contusive injury in rats can eliminate supraspinally mediated responses to below-level test stimuli (Baastrup et al., 2010; Bedi et al., 2010), raise questions about the extent to which below-level pain is driven by below-level neural activity in many rodent SCI models. In addition to potentially promoting below-level pain after incomplete SCI, below-level central sensitization driven by nociceptor activity is likely to contribute to below-level somatic and autonomic hyperreflexia, such as sensitized responses of hindlimbs to mechanical and thermal test stimuli (Waxman and Hains, 2006; Hulsebosch et al., 2009; Yezierski, 2009) or hypertensive autonomic dysreflexia (Rabchevsky, 2006). Some DRG neurons innervate the viscera and most of these meet the functional definition of nociceptor (Christianson and Davis, 2010; Gold and Gebhart, 2010). Enhanced activity occurring in visceral nociceptors after SCI might contribute to visceral pain (Siddall et al., 2003; Kogos et al., 2005) and other visceral problems, such as bladder and gastrointestinal dysfunction (de Groat and Yoshimura, 2011; Fynne et al., 2012). Below-level somatic, autonomic, and visceral effects of nociceptor activity are likely to be enhanced by hyperexcitability of spinal circuits resulting from disinhibition consequent to interruption of descending inhibitory tracts by SCI (Lu et al., 2008) (Figure 1).

At-level alterations of nociceptors (Figure 1) are well situated for driving spontaneous and evoked at-level pain by direct excitation of pain pathways and promotion of at- and above-level central sensitization. Direct and indirect effects of injury on many other cell types within the damaged region of the cord are also likely to contribute to at-level pain (e.g., Yezierski, 2009). Above-level neuropathic pain in human patients is much less common than at- and below-level pain, although above-level reflex hypersensitivity is readily produced in rodents (Carlton et al., 2009; Densmore et al., 2010). An interesting possibility is that somal SA in nociceptors, which in rats has been observed at and below but not far above the injury level (Bedi et al., 2010), is more important for driving conscious pain than is peripherally generated SA, which occurs in nociceptors far above the injury level (Carlton et al., 2009). Consistent with this possibility, firing rates observed in nociceptors after SCI are substantially higher for somal SA (~1 Hz) (Bedi et al., 2010) than peripheral SA (<0.1 Hz) (Carlton et al., 2009). This would suggest that forelimb hypersensitivity may occur with relatively little ongoing pain in the forelimb, consistent with growing evidence that hyperreflexia and conscious pain in rodents, as in humans, can be poorly correlated after SCI (Baastrup et al., 2010). It will be important to see if somal SA in nociceptors is correlated with operant measures of SCI pain associated with different spinal levels in rats. The correlation Bedi et al. (2010) found between SA in nociceptors sampled at but not below a contusion site with vocalization—a supraspinal response that is sometimes linked to aversive behavior in rodents (e.g., Meagher et al., 2001)—suggests that at-level somal SA might help to drive conscious pain in SCI rats.

If SA (somally and peripherally generated) and other hyperfunctional alterations in nociceptors contribute significantly to human at- and below level pain, as well as to hyperreflexia and visceral problems after SCI, pharmacological agents that selectively target nociceptor hyperexcitability should be clinically useful. A number of genes important for excitability are preferentially expressed in nociceptors, raising the possibility that blocking their function could ameliorate some of the suffering caused by SCI while producing minimal side effects. Indeed, preliminary results show that SA in dissociated nociceptors depends upon nociceptor-specific Nav1.8 channels, and indicate that knocking down the expression of these channels can attenuate behavioral hypersensitivity after SCI (Yang et al., 2012). As efforts to develop effective *in vivo* blockers of Nav1.8 channels and other nociceptor-specific ion channels proceed, such drugs may become useful for treating chronic pain in SCI and other conditions involving hyperfunctional nociceptors.

### Conflict of interest statement

The author declares that the research was conducted in the absence of any commercial or financial relationships that could be construed as a potential conflict of interest.
